# *Heteropelta boboi* n. gen., n. sp. an armored archosauriform (Reptilia: Archosauromorpha) from the Middle Triassic of Italy

**DOI:** 10.7717/peerj.12468

**Published:** 2021-11-15

**Authors:** Fabio Marco Dalla Vecchia

**Affiliations:** Mesozoic Research Group, Institut Català de Paleontologia Miquel Crusafont (ICP), Sabadell, Catalonia, Spain; Museo Friulano di Storia Naturale, Udine, Italy

**Keywords:** Reptiles, Archosauriformes, Dorsal armor, Osteoderms, Middle Triassic, Alps, Italy

## Abstract

*Heteropelta boboi* is a new archosauriform reptile from the upper Anisian of northeastern Italy represented by a fragment of dorsal armor with a row of neural arches of the dorsal vertebrae. The dorsal armor of the new taxon is composed of two columns of paramedian osteoderms and at least six columns of lateral osteoderms. Unlike other armored archosaurs, the osteoderms are imbricated with the posterior osteoderm overlapping the anterior one. The low neural arches bear small neural spines and long postzygapophyses. The osteoderms of the lateral columns increase in size and change in shape from the most medial to the most lateral columns. Among the Archosauriformes, only the non-archosaur proterochampsians *Vancleavea campi*, *Litorosuchus somnii*, and the doswelliids have dorsal armor comprised of more than two columns of osteoderms per side, but the morphology and arrangement of their osteoderms is unlike those of the new Italian taxon. A cladistic analysis of Archosauromorpha positions *Heteropelta boboi* as either a basal phytosaur or a basal suchian. However, a second cladistic analysis focused on armored archosaurs alternatively positions the new taxon as a basal archosauriform, basal suchian, basal loricatan or crocodylomorph. Better resolution of the phylogenetic relationships of *Heteropelta boboi* will likely be obtained only with the discovery of cranial and postcranial remains associated with its diagnostic armor elements.

## Introduction

Since the beginning of this century, the Middle Triassic Torbiditi d’Aupa Formation (Aupa Turbidites, TAF) of the eastern Carnic Alps (NE Italy) has begun to yield abundant reptile remains belonging to tanystropheids, archosauriforms, sauropterygians and ichthyosaurs (Dalla Vecchia, 2006, 2008).

**Figure 1:**
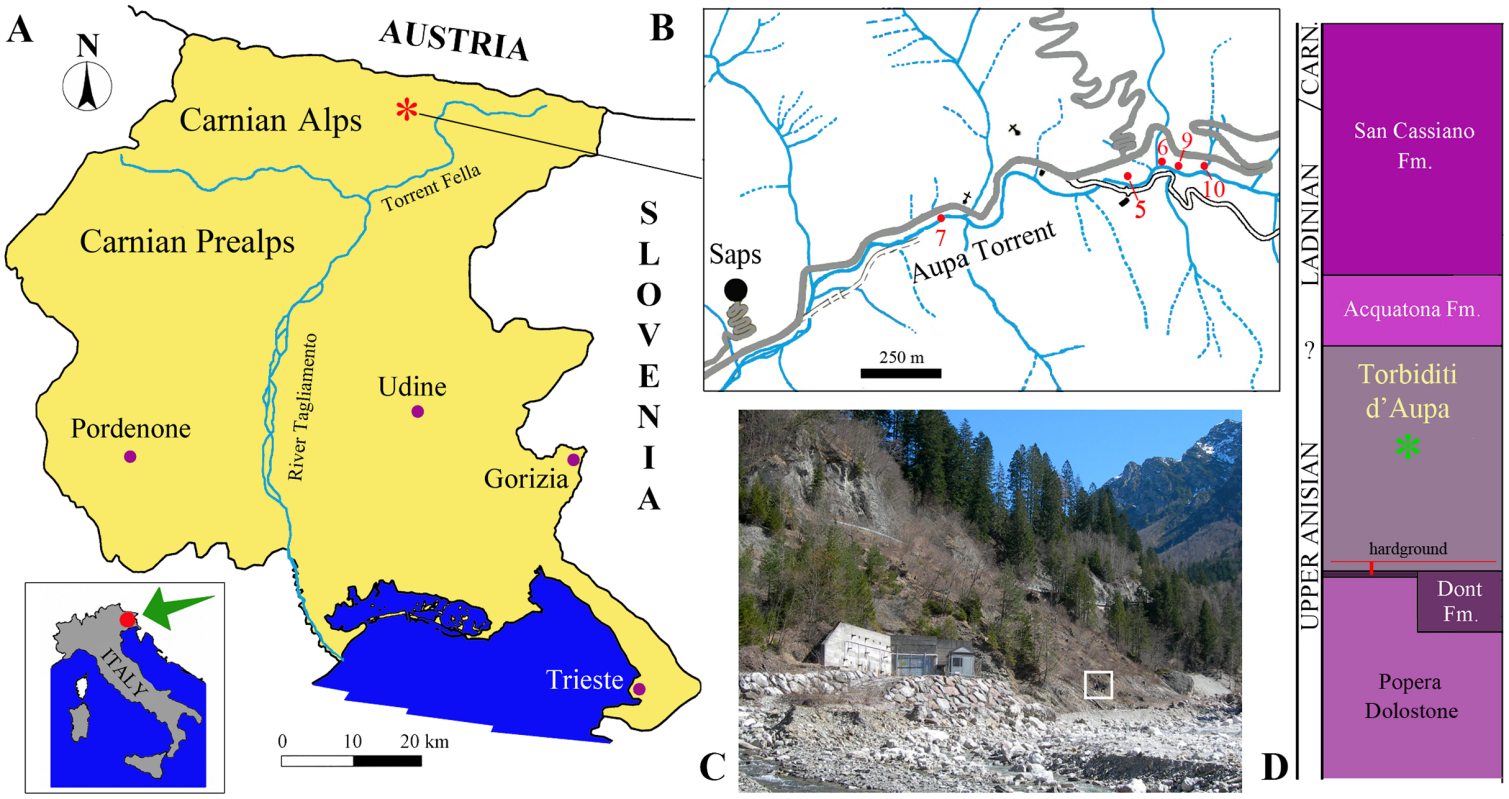
Locality and stratigraphic provenance of the specimens. (A) Location of the Aupa Valley in the Friuli Venezia Giulia Autonomous Region of NE Italy; (B) Location of the fossil-bearing outcrops (*sensu* Dalla Vecchia & Simonetto, 2018) mentioned in the text; (C) photo of the outcrops (outcrop 9 corresponds approximately with the square); (D) position of the specimens (asterisk) within the Torbiditi d’Aupa Formation and the local stratigraphic section.

These remains were found in a small area along the Aupa Creek near Saps village (Moggio Udinese Municipality, Friuli Venezia Giulia Autonomous Region, Trieste, Italy; Figs. 1A–1B). They come from 10 distinct outcrops (see Dalla Vecchia, 2006, 2010; Dalla Vecchia & Simonetto, 2018). Although close to each other, these outcrops may represent different levels within the TAF, as suggested by the different lithologies of the specimens.

The TAF (Fig. 1D) is 350 m thick in the Aupa Valley (Jadoul & Nicora, 1979), but the vertebrate remains probably occur only in one or two relatively thin horizons within the formation that represent regressive events (Farabegoli, Jadoul & Martines, 1985, p. 169). It is difficult to know the exact thickness of the fossil-bearing horizons and the stratigraphic relationships of the ten fossil-bearing outcrops, because of the strong tectonic distortion and the absence of detailed studies. No detailed stratigraphic correlation of the fossiliferous outcrops has been made to date.

The TAF is dated to the late Anisian-Anisian/Ladinian boundary by Jadoul & Nicora (1979, p. 14–15) and to the latest Anisian (late Illyrian) by Farabegoli, Jadoul & Martines (1985). The ammonoid *Hungarites* gr. *zalaensis*, which was collected from outcrop 6 (Dalla Vecchia, 2006) ranges from the upper Anisian**-**lower Ladinian, and is common in the upper Anisian. Therefore, the TAF is approximately coeval with the Grenzbitumenzone/Besano Formation of Switzerland and Italy, which yielded a reptile association that also include the long-necked tanystropheid *Tanystropheus*, archosauriforms (*Ticinosuchus ferox*), a large eusauropterygian *sensu* Rieppel (2000) (*Nothosaurus giganteus*) and ichthyosaurs (Rieppel, 2019).

The depositional environment of the TAF has been considered as deep marine (Jadoul & Nicora, 1979; Farabegoli, Jadoul & Martines, 1985; Nicora & Rizzi, 1998), as emphasized by the use of the word “turbidites” in the name of the formation. However, the bone-bearing horizons are the result of the progradation of fan deltas into the basin during the acme of the regressive cycles, according to Farabegoli, Jadoul & Martines (1985). The fan deltas developed at the margin of the Anisian Paleocarnic Ridge, which was an emergent area located just north of the present day Aupa Valley (Farabegoli, Jadoul & Martines, 1985, fig. 11).

Among the reptile specimens is a partial axial skeleton with armor (MFSN 46485) that has no correspondence in the known fossil record. Here, this specimen is referred to a new genus and species, and isolated osteoderms found in the same formation and locality are described and compared with those of MFSN 46485 in order to establish their affinity with the new taxon.

## Materials, methods and terminology

The reptile remains found in the TAF are represented by isolated bones and teeth that often show evidence of breakage, damage and transport. The sample consists of about 500 skeletal remains, 178 of which are referred to as “indeterminate reptile” (Dalla Vecchia & Simonetto, 2018) because they are fragmentary, still unprepared and/or not adequately studied yet. Most of the identifiable bones have been preliminarily referred to the peculiar tanystropheid *Tanystropheus* (over 200 specimens, belonging to large-sized individuals); the other identified bones belong, in order of decreasing frequency, to archosauriforms (36 to possibly 44 specimens), a eusauropterygian *sensu* Rieppel (2000) (probably a large-sized *Nothosaurus* species; 38 specimens), a medium-sized ichthyosaur (two specimens), and a cyamodontoid placodont (one armor fragment) (Dalla Vecchia, 2006, 2008, 2010; Dalla Vecchia & Simonetto, 2018). Fish remains are restricted to a couple of bones of a large coelacanthiform (Dalla Vecchia, 2008). Fragments of terrestrial plants are sometimes associated with the bones. Outcrop 6 also yielded a few bivalves and an ammonoid (Dalla Vecchia, 2006).

**Figure 2:**
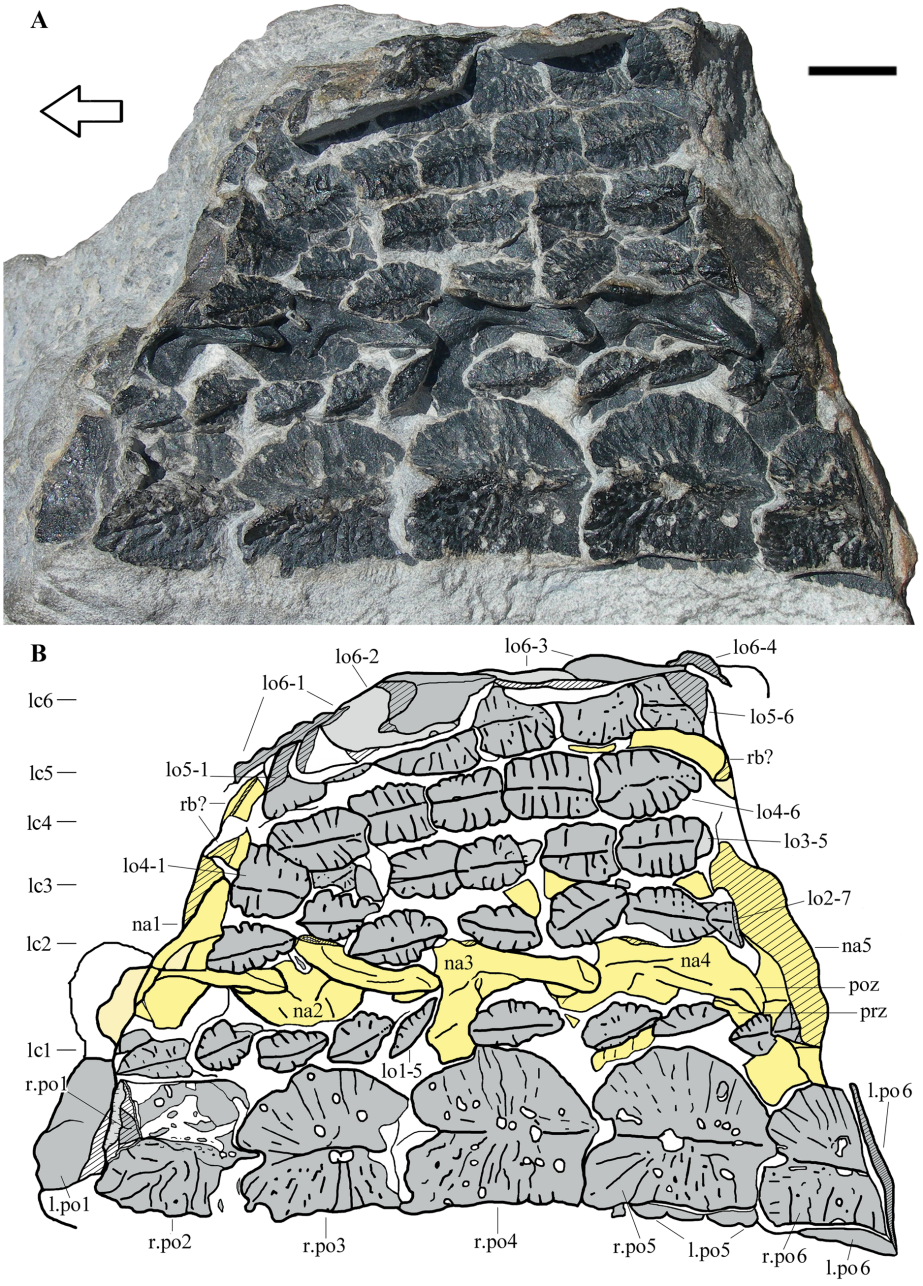
*Heteropelta boboi*, MFSN 46485, holotype. (A) The specimen in external view; (B) Drawing of the specimen. Abbreviations: l., left; lo, lateral osteoderm; lc1–6, longitudinal columns of lateral osteoderms (lc6 is the most lateral column); na, neural arch; po, paramedian osteoderm; poz, postzygapophysis; prz, prezygapophysis; r., right; rb, rib. Osteoderms are numbered progressively from the most anterior to the most posterior of each column; neural arches are numbered progressively from the most anterior to the most posterior. Osteoderms are in gray color, the impression of the osteoderms (where all or most of the bone has split away) is in pale grey, neural arches and ribs are in yellow (pale yellow where most of the bone has split away), and rock is white. Lined pattern indicates broken bone and rock. Arrow points to the anterior (cranial) direction. Scale bar equals 10 mm.

The new taxon described here is based on a fragment of dorsal armor (MFSN 46485) which is preserved in a small block of dark gray sandstone rich in plant macroremains (Fig. 2). The specimen was found *in situ* in outcrop 9 (Figs. 1B and 1C).

Twenty-one isolated osteoderms (MFSN 31568-70, 31577, 31582, 34991, 46614-46618 and 46828-37) are compared with MFSN 46485. Two of these osteoderms (MFSN 46614 and 46615) are from the same outcrop (9) as MFSN 46485; four from outcrop 5 (MFSN 46616-18 and 46835); eight from outcrop 6 (MFSN 31568-69, 31582, 37580, 31577, 34991 and 46833-34); two from outcrop 7 (MFSN 46836-37); and five from outcrop 10 (MFSN 46828-32). The GPS locations of the outcrops are on file with the MFSN.

The reference for the definition of the zygapophyses is Romer (1956, p. 225). The anteroposterior polarity of the specimen MFSN 46485 is based on the position of the prezygapophyses and postzygapophyses in the articulated neural arches. The term “paramedian osteoderms” is commonly reported in the descriptions of archosauriform reptiles, but it is used to indicate both a single longitudinal row of osteoderms and two rows. The meaning of the adjective “paramedian” given by the Merriam-Webster Medical Dictionary (https://www.merriam-webster.com/medical/paramedian) is “situated adjacent to the midline”. Therefore, paramedian osteoderms are only those arranged into two parallel longitudinal rows that contact each other along the midline of the body. One of the reviewers recommended the use of the term “column” instead of “row” to indicate the longitudinal rows of osteoderms in the armor, following the recent works on aetosaurs (*e.g*., Desojo et al., 2013). Therefore, from here on I use the word “column” in the text instead of “row” for longitudinal rows; transverse bands are still referred to as “rows”.

The references as for phylogenetic relationships among archosauromorphs are Nesbitt (2011), Ezcurra (2016) and Ezcurra et al. (2020).

In order to assess its phylogenetic position, *Heteropelta boboi* has been coded in the matrices of Ezcurra et al. (2020) and Marsh et al. (2020). The phylogenetic analysis by Ezcurra et al. (2020) is focused on the relationships within the Archosauromorpha and includes 157 terminal taxa plus the new one (see Supplemental Information). Because of the incompleteness of the specimen, only 14 out of 822 characters were scored for *H. boboi* (1.72% of the parsimony-informative characters; one character is constant and 11 variable characters are parsimony-uninformative).

The phylogenetic analysis by Marsh et al. (2020), which includes 91 terminal taxa plus the new one, is focused on the relationships among armored archosaurs. With respect to the matrix by Ezcurra et al. (2020), it contains some more armored taxa (*e.g*., *Acaenasuchus geoffreyi*, *Revueltosaurus callenderi*, *Euscolosuchus olseni* and 13 crocodylomorphs) and several characters specific to osteoderm morphology and arrangement. The incompleteness of MFSN 46485 allowed for scoring of only 9 out of 445 characters for *H. boboi* (2.08% of the parsimony-informative characters; two characters are constant and 11 variable characters are parsimony-uninformative). Several characters regarding the osteoderms are not applicable to the new taxon.

The modified matrices were used to perform parsimony-based phylogenetic analyses by TNT 1.5 (Goloboff & Catalano, 2016). The analyses used 100 ‘New Technology’ search replicates by default settings, saving all the shortest trees inferred. Subsequently, the ’Traditional Search’ heuristic search analyses exploring the tree islands inferred by the first round of analyses was performed for each analysis.

In the analysis by Marsh et al. (2020), the Phytosauria fall outside the Archosauria, whereas they fall inside Archosauria as early-diverging pseudosuchians in the analysis by Ezcurra et al. (2020).

The electronic version of this article in portable document format will represent a published work according to the International Commission on Zoological Nomenclature (ICZN), and hence the new names contained in the electronic version are effectively published under that Code from the electronic edition alone. This published work and the nomenclatural acts it contains have been registered in ZooBank, the online registration system for the ICZN. The ZooBank Life Science Identifiers (LSIDs) can be resolved and the associated information viewed through any standard web browser by appending the LSID to the prefix http://zoobank.org/. The LSID for this publication is: urn:lsid:zoobank.org:pub:B91F17BF-7B38-4034-B761-170916EBA16E. The online version of this work is archived and available from the following digital repositories: PeerJ, PubMed Central and CLOCKSS.

## Results

**SYSTEMATIC PALEONTOLOGY**

Reptilia Laurenti, 1768

Diapsida Osborn, 1903

Archosauromorpha von Huene, 1946

Archosauriformes Gauthier, Kluge & Rowe, 1988

*Heteropelta* gen. nov.

**ZooBank.** urn:lsid:zoobank.org:act:8F8B8564-B2E6-4B7D-B2E1-14BDFAB36900

*Heteropelta boboi* sp. nov.

(Figs. 2–4)

**Figure 3:**
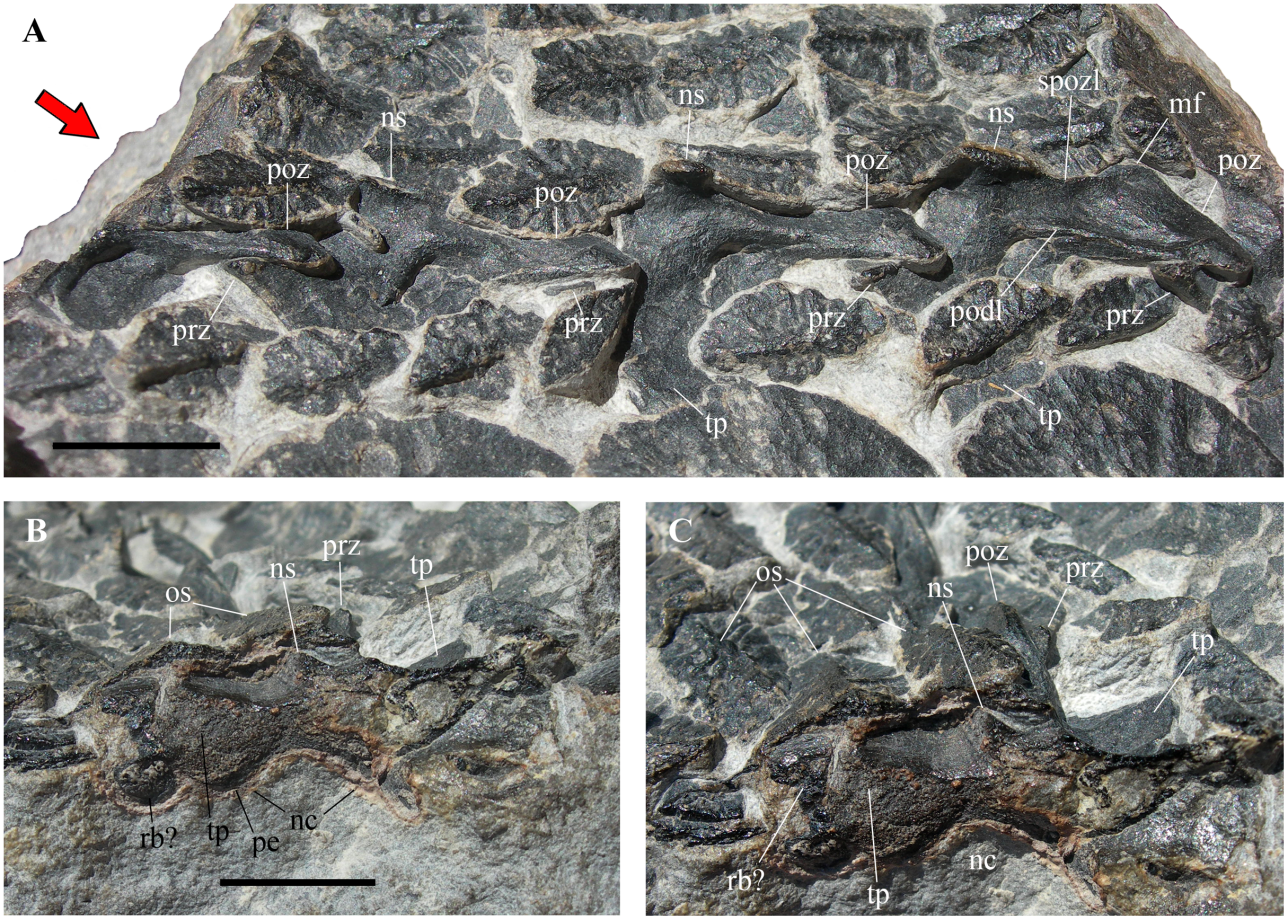
*Heteropelta boboi*, MFSN 46485, holotype, neural arches. (A) The articulated neural arches in left dorsolateral view; (B) diagonal cross section of the most anterior neural arch (na1 of Fig. 2) in anterior view (red arrow in A); (C) same as (B), but in anterodorsal view. Zygapophyses are all from the left side. Abbreviations: mf, medial flange of the postzygapophysis; nc, neural canal; ns, neural spine; os, osteoderm of the lateral columns; pe, pedicel; podl, postzygodiapophyseal lamina; poz, postzygapophysis; prz, prezygapophysis; rb, rib; spozl, spinopostzygapophyseal lamina; tp, transverse process. The anterior (cranial) direction is to the left in (A). Scale bar equals 10 mm.

**Figure 4:**
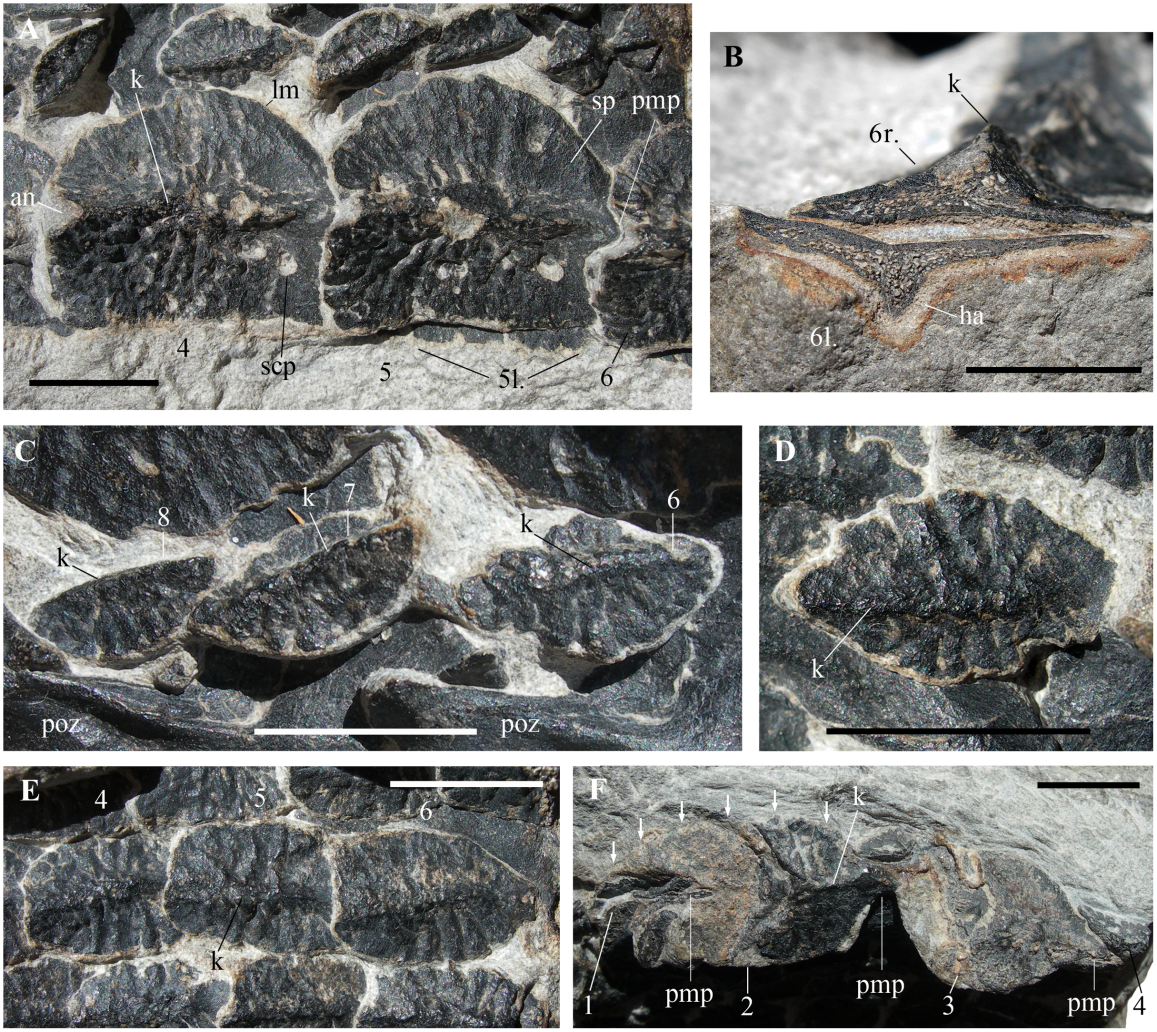
*Heteropelta boboi*, MFSN 46485, holotype, details of the osteoderms. (A) The right paramedian osteoderms 4 and 5 (a small portion of the left paramedian osteoderm 6 is also visible); (B) cross section of the right (above) and left (below) paramedian osteoderms 6; (C) osteoderms 6–8 of the first lateral column (lc1 in Fig. 2B); (D) detail of osteoderm 3 of the second lateral column (lc2 in Fig. 2B); (E) three imbricated osteoderms of the fourth lateral column (lc4 in Fig. 2B; osteoderms lo4-4 to 6); and (F) the remains of the five osteoderms of the most lateral column (lc6 in Fig. 2B) (arrows point to the right lateral margin of the best preserved element, lo6-2 of Fig. 2B). Abbreviations: 1–8, progressive numbers of the osteoderms along each longitudinal column, from the most anterior to the most posterior; an, anterior notch of the paramedian osteoderm; ha, halo surrounding the cross-sectioned paramedian osteoderm; k, keel; l., left; lm, lateral margin of the paramedian osteoderm; pmp, posterior median process; poz, postzygapophysis; r., right; scp, subcircular pit of the paramedian osteoderm; sp, smooth portion of the paramedian osteoderm. In (A), (D), and (E–F), the anterior (cranial) direction is to the left, whereas it is to the right in (C). All scale bars equal 10 mm.

**ZooBank.** urn:lsid:zoobank.org:act:BCADA4F4-47FA-448B-B71D-6D733AD3AACE

**Etymology**. *heteros* (ἕτερος), Greek for “different” and *pelte* (πέλτη), Greek for “shield”, in reference to the differentiated osteoderms of its armor; *boboi* after the nickname of the finder, Mr. Roberto “Bobo” Rigo.

**Holotype**. MFSN 46485, partial armor made of eight columns of osteoderms and five neural arches of dorsal vertebrae (Fig. 2).

**Referred specimens** *—*

**Stratigraphic horizon and locality.** Torbiditi d’Aupa Formation, uppermost Anisian (upper Illyrian), Middle Triassic (Fig. 1C); outcrop 9 of Dalla Vecchia & Simonetto (2018), Aupa Valley near Saps village, Moggio Udinese municipality, Udine Province, Friuli Venezia Giulia Autonomous Region, Italy.

**Diagnosis.** Armored archosauriform with the following unique combination of characters (autapomorphies are indicated with an asterisk): low neural arches of the dorsal vertebrae with small, transversely thin and craniocaudally very short neural spines and long postzygapophyses; dorsal armor composed of two columns of paramedian osteoderms and at least six columns of lateral osteoderms; lateral columns arched with respect to the paramedian columns; paramedian osteoderms are square, with a straight medial margin and subcircular lateral margin; external surface of the paramedian osteoderms with a tall longitudinal median keel and coarse ornamentation of pits, grooves and ridges except in the smooth posterior portion; ornamentation is more marked on the medial side of the osteoderm; paramedian osteoderms are imbricated, with a posterior median process matching an anterior median concavity on the internal side of the following osteoderm (the opposite respect to the usual articulation of paramedian osteoderms)*; the anterior margin of the paramedian osteoderm is notched in correspondence to the anterior end of the keel where the posterior median process of the preceding paramedian osteoderm articulates; osteoderms of the lateral columns increase in size and change in shape from the most medial to the most lateral columns*; osteoderms of lateral columns 1–5 with a median longitudinal keel and ornamented with ridges and grooves perpendicular to the keel; osteoderms of lateral columns 1–2 with elliptical outline and pointed anteroposterior extremities; osteoderms of lateral columns 4–5 have a similar outline as those of columns 1–2 but their anterior margin is not pointed and is perpendicular to the keel; osteoderms of lateral column 3 intermediate in shape; osteoderms of lateral column 6 much larger than those of the other lateral columns, anteroposteriorly longer than mediolaterally wide, sub-pentagonal or ‘arrowhead-shaped’ and with a median posterior process; osteoderms of lateral columns 3–6 are imbricated with the posterior element overlapping the anterior one*.

**DESCRIPTION OF THE HOLOTYPE**

MFSN 46485 is a 105 mm long and 75 mm wide fragment of armor composed of eight columns of osteoderms (Fig. 2). Two paired columns at the left extremity of the armor fragment (lower extremity in Fig. 2) are made of osteoderms that are markedly larger than those of the adjacent five columns and have a different shape. The left one of these two columns is folded below the other and its osteoderms can be seen mostly at the extremities of the rock fragment as cross sections (Fig. 2). A row of five still articulated neural arches that is parallel to the columns of osteoderms crops out between the first and the second columns of lateral osteoderms (Fig. 2).

**Neural arches**

The five articulated neural arches (numbered 1 to 5 in anteroposterior direction) expose their left dorsolateral side (Figs. 2 and 3A). The right side is covered by the second column of lateral osteoderms and by matrix. The line of neural arches is displaced to the right with respect to its probable anatomical position below the two paired columns of larger osteoderms; it has broken the alignment of the osteoderms of the lateral columns 1 and 2.

The arches are unfused to their centra, thus MFSN 46485 may belong to an immature individual. Four left zygapophyseal articulations are well exposed, allowing the unambiguous identification of pre- and postzygapophyses and the anteroposterior polarity of the whole specimen, under the assumption that a perfect 180° rotation of the vertebral column with respect to the dorsolateral armor is unlikely.

The neural arches are low (Fig. 3). Lengths measured between the extremities of the zygapophyses are 25 mm in arches 2 and 3 and 26 mm in arch 4. The distance from the neural spine to the lateral-most point of the postzygapophysis is 3.5, 2.8 and 4.5 mm in arches 2, 3 and 4, respectively. The pedicels (visible as diagonal cross sections in arches 1 and 5 at the extremities of the row; Figs. 3B–3C) appear to be rather low and barely distinct from the transverse processes. The latter are never fully exposed; they appear to be massive in anterior view (Figs. 3B–3C) and relatively long and projecting laterally from the neural arch in dorsal view (Fig. 3A).

The neural spines are broken, but their basal part is preserved in neural arches 2–4. The spines are very short anteroposteriorly and much more narrow transversely; that of the central arch 3 is shorter than the other two, being only two mm long anteroposteriorly, whereas that of the following arch (4) has a minimum anteroposterior length of four mm and a transverse width of scarcely one mm. The cross section of the base of spine 2 is about five mm long and about one mm wide. The preserved portion of the neural spine of arch 3 is only four mm high, but it is constricted in the middle in lateral view and expands fan-wise to four mm long apically, suggesting that the complete spine was not much taller. There is a thin spinopostzygapophyseal lamina. As the zygapophyses are articulated and the postzygapophysis has slightly shifted posteriorly in all arches, the prezygapophyses are partially covered by the postzygapophyses and by osteoderms of the first lateral column as well. Both zygapophyses are slightly splayed laterally and the postzygapophysis is much longer than the prezygapophysis (more than twice as long, possibly three times longer in neural arch 4); the postzygapophyses increase in length posteriorly, measuring 12.5, 13, 14 and 15 mm long in neural arches 1 to 4 respectively. The prezygapophyses are short and stout, with expanded articular ends, and placed rather laterally with respect to the neural spine (they seem to project from the base of the transverse process). The postzygapophyses are elongate and have an unusual triangular outline in dorsal view due to the presence of a medial flange (Fig. 3A). There is a ridge-like postzygodiapophyseal lamina.

The prezygapophyseal facets face medially and slightly dorsally whereas the postzygapophyseal facets face lateroventrally; the articulation plane is inclined at about 70°.

Fragments of dorsal ribs are possibly preserved on the right side of the neural arches 1 and 5 (Fig. 2).

**Osteoderms**

All osteoderms, excluding those visible in cross-section and those at the left extremity of the armor fragment, are exposed in external (dorsal) view. They have an ornamented external surface and a longitudinal median keel. The anteroposterior polarity of the osteoderms is inferred based on the orientation of the neural arches, although this is counter-intuitive in comparison with the condition in other archosauriforms (see Discussion).

The two columns of larger medial osteoderms are made of six elements for each column (Fig. 2). The right column is exposed whereas its antimere is rotated 180° and lies below it inside the rock. They are paramedian osteoderms (see Discussion). The first and last elements of the columns are incomplete and reveal their cross-sections. Only a posterior fragment of the first osteoderm of the right column is preserved; it is fitted inside the following second osteoderm, which is anteriorly damaged. Like the neural arches, the paramedian osteoderms are numbered 1 to 6 in anteroposterior direction for ease of description. Osteoderms 3–5 of the right column are complete and measure 19, 22 and 22 mm in length and 17, 19 and 19.5 mm in width, respectively. Therefore, they have about equal dimensions, being slightly longer than wide. There is a prominent longitudinal median keel extending along the entire anteroposterior length of the osteoderm (Figs. 2 and 4A–4B). The two halves of the osteoderm separated by this keel have a different outline: the medial half is rectangular, longer than wide, with a straight medial margin along the contact with its antimere, whereas the lateral half is semicircular, with a curved lateral margin that becomes thicker in the posterior tract where it meets the keel. The lateral half is not ventrally angled respect to the medial one and the osteoderm is nearly flat (Figs. 2 and 4A–4B). The ornamentation of the medial half of the osteoderm is composed of thick and irregular branching ridges separated by grooves; ridges and grooves are more or less perpendicular to the keel in the central part and slightly radial at the anterior extremity. The lateral half of the osteoderm is less sculptured than the medial one and shows a more regular pattern of thinner radial ridges (Fig. 4A). There are also large subcircular pits, which are irregularly distributed. The posterior fourth of the osteoderm is smooth, showing only some subcircular pits.

The paramedian osteoderms are imbricated. Imbrication takes places by means of the posterior prolongation of the keel in the form of a posteriorly projecting and wedge-like process (which is not fully visible because the osteoderms are articulated) that fits ventrally in a deep concavity on the anterior end of the internal surface of the following osteoderm (the apex of the keel of the anterior osteoderm fits in the apex of the concavity of the posterior osteoderm). The posteriormost part of the osteoderm body is also overlapped by the anteriormost part of the following osteoderm. Thus, the anterior part of the osteoderm has the cross-section of an upside-down V and receives the posterior portion of the preceding osteoderm, which has a complementary shape; this can be seen in the cross-section of the anterior portion of the right paramedian osteoderm 2, which shows the posterior end of the preceding osteoderm inside. In dorsal view, the anterior margin of the osteoderms is notched in correspondence of the anterior end of the apex of the keel.

These paramedians are thick in correspondence of the tall median keel, whereas the medial and lateral halves are thin and taper toward the outer margin (Fig. 4B). The inner part of the osteoderm is spongy; the compacta is thin (Fig. 4B). The cross-sectioned osteoderms six are surrounded by a light halo contoured by a reddish iron oxides film (Fig. 4B). This halo was probably originated by the decay of a non-mineralized cover of the osteoderms (Brown, 2017).

The paramedians are aligned one-to-one with the corresponding neural arches and are not staggered.

The six lateral columns of osteoderms are parallel to each other. Lateral columns 1 and 2 are disturbed by the emergence of the neural arches; undisturbed lateral columns 3–5 are slightly arched in dorsal view and appear to converge anteriorly toward the right column of paramedian osteoderms. The lateral osteoderms do not appear to be arranged in transverse rows, but this could be due to the slight disarticulation of the columns.

The first five lateral columns are composed of elliptical osteoderms that are much smaller than those of the paramedian columns (Fig. 2). The lateral columns 1–5 preserve the remains of 10, 7, 5, 6, and 6 osteoderms, respectively. The sixth and most lateral column is composed of larger, sub-pentagonal or ‘arrowhead-shaped’ osteoderms, which are comparable in length to the paramedian osteoderms but are narrower.

Osteoderm size increases laterally, *i.e*., osteoderms of the first lateral column are about 10 mm long, those of the second column are about 12 mm long and those of the fourth column are 13–14 mm long. The most lateral (6th) column is made of even larger osteoderms. The osteoderms of the first two lateral columns have pointed anterior and posterior ends. Although maintaining their anteroposterior alignment, they are slightly disarticulated and do not show imbrication. Osteoderms of columns 3–5 have an overall shape that is similar to that of the osteoderms of columns 1–2 but their anterior portion is not pointed and the posterior osteoderm overlaps the anterior one dorsally with a kind of articulation resembling that of the paramedian osteoderms (Fig. 4E).

The osteoderms of the lateral columns 1–5 all have the same ornamentation: a longitudinal median keel dividing the osteoderm into two symmetrical halves and thick ridges that are perpendicular to the keel or slightly radial and are separated each other by narrow grooves (Figs. 4C–4E).

The most external osteoderm column on the right side of the specimen (lateral column 6) preserves the remnants of four articulated elements (Fig. 4F). These osteoderms are arranged nearly perpendicularly to those of the other columns and their dorsal sides face laterally or laterodorsally with respect to the overall orientation of the specimen. It is unclear whether this is the anatomical orientation of the osteoderms or it is caused by taphonomic factors. In the first case, the column would probably represent the lateral termination of the armor. None of the osteoderms of the lateral column 6 is completely preserved and the first and last elements are just small fragments. The two most complete osteoderms preserve the posterior part as bone and the incomplete anterior part as impression. They have a long median posterior process fitting below the following osteoderm like the paramedian osteoderms, a median longitudinal dorsal keel and a body that is longer than wide (Fig. 4F). The best preserved osteoderm of this column (osteoderm 6–2 of Fig. 2) is 26 mm long, including the posterior process, and 15 mm wide.

**DESCRIPTION OF ISOLATED OSTEODERMS**

The tetrapod sample from the TAF includes 21 isolated osteoderms. They are compared to those preserved in MFSN 46485, in order to see if they can be assigned to *Heteropelta boboi* based on their size and morphology.

**Figure 5:**
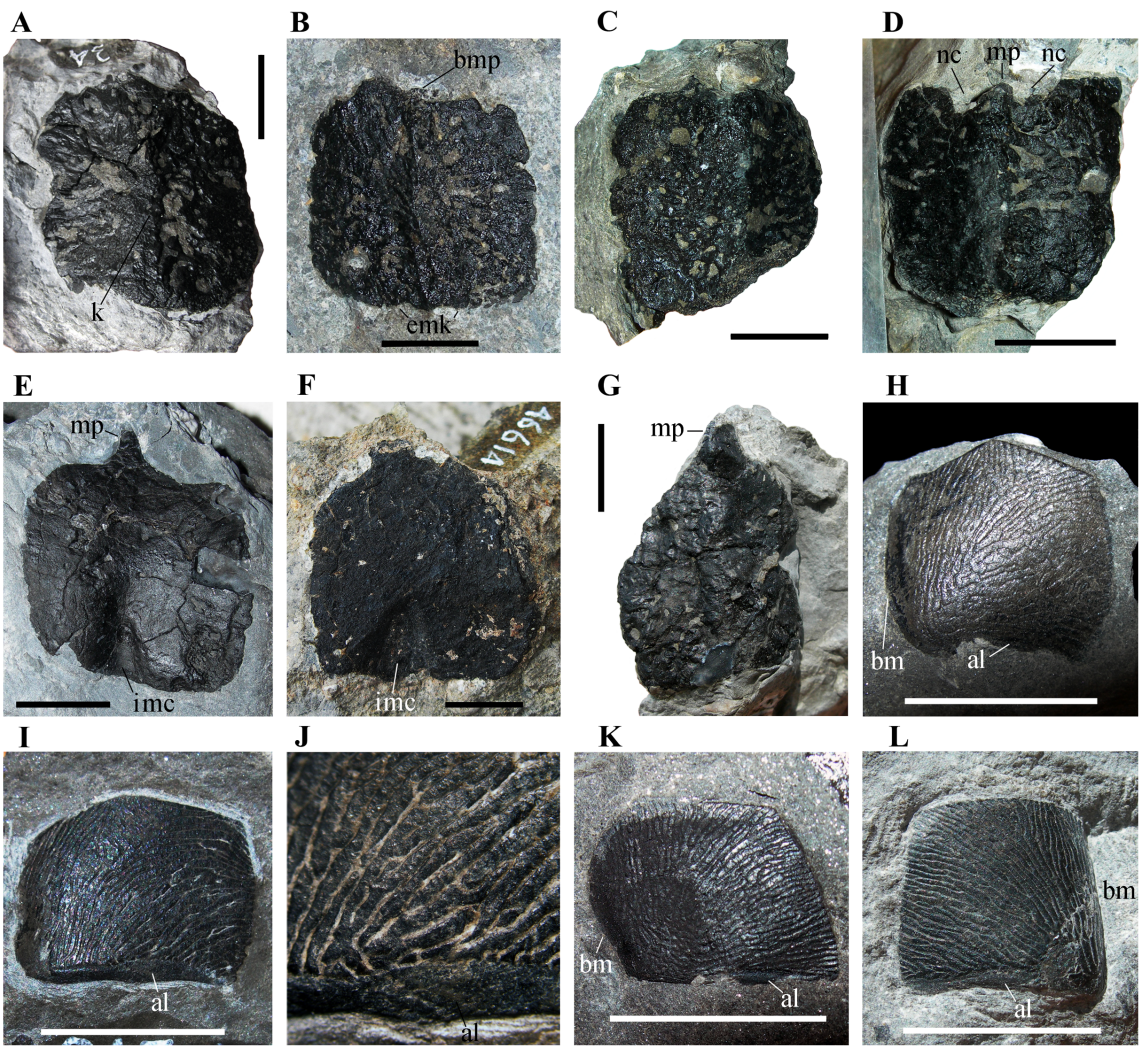
Isolated osteoderms from the Torbiditi d’Aupa Formation of the Aupa Valley. (A) MFSN 31577; (B) MFSN 46615; (C) MFSN 46616; (D) MFSN 46617; (E) MFSN 34991; (F) MFSN 46614; (G) MFSN 46618; (H) MFSN 46829; (I) MFSN 31568; (J) close-up of MFSN 31568; (K) MFSN 46828; and (L) MFSN 46833. Abbreviations: al, articular lamina; bm, bent margin; bmk, broken median process; emk, enlargement of the median keel; imc, internal median concavity; k, longitudinal median keel; mp, median process; nc, notch lateral to the median process. (A–D), (G) and (H–L) are in external view, whereas (E–F) are in internal view. All scale bars equal 10 mm.

Seven specimens (MFSN 31577, 37570, 34991 and 46614-46617; Figs. 5A–5F) are square in outline like the paramedian osteoderms of MFSN 46485 and are slightly longer than wide, excluding MFSN 46617 that is slightly wider than long. Their size ranges from 18–31 mm in length (as preserved, *i.e.*, mostly lacking the median process) and 19–28 mm in width. These osteoderms are nearly all preserved in coarse arenite and have worn margins; for this reason, the pointed median process that represents the termination of the longitudinal median keel is broken or missing in six out of seven specimens. The pointed process is completely preserved only in MFSN 34991 (Fig. 5E) and partly in MFSN 46617 (Fig. 5D).

Four out of seven osteoderms are exposed in external view (Figs. 5A–5D), whereas three (MFSN 34991, 46614 and 46679; Figs. 5E–5F) are in internal view.

All osteoderms exposed in external view have a longitudinal medial keel and a coarse ornamentation made of irregular pits, ridges and grooves (Figs. 5A–5D). The keel is low and blunt in all specimens except MFSN 46615, which has a sharper keel that is transversely enlarged at one extremity (Fig. 5B), probably corresponding to the median concavity of the internal side. The two halves separated by the keel appear to have a similar shape and size, at least in the most complete osteoderms. These two halves meet at a lower angle (ca. 115°) in MFSN 46615 and MFSN 46617 than in the other larger osteoderms; MFSN 46615 also differs in having a concave internal face. MFSN 46617 has two small notches lateral to the incomplete median process (Fig. 5D). In all specimens, the ornamentation is variably affected by abrasion due to transport and preservation in coarse sediment. In most osteoderms, the elongated pits and ridges show a hint of a radial pattern.

The internal side is smooth and nearly flat in MFSN 34991 and 46614 (Figs. 5E–5F); at one extremity there is a median concavity (a notch with a triangular outline) for the median process of the opposite side of the adjacent osteoderm.

One osteoderm exposed in external view (MFSN 46618, 26 mm long and 18.5 mm wide; Fig. 5G) has a subtriangular or drop-like outline, with the longer lateral margins converging to the pointed median process that represents the termination of the longitudinal median keel. The margin opposite to the process is straight and nearly perpendicular to the median keel. The keel is low and blunt. The ornamentation is coarse and made of irregular pits, ridges and grooves. This osteoderm is robust; it appears to be nearly three millimeters thick along one of the lateral margins. It resembles the arrowhead-shaped cervical to mid-caudal paramedian osteoderms of *Ticinosuchus ferox* (see Krebs, 1965, figs. 8–12, 16, and 61; Lautenschlager & Desojo, 2011) and the heart- or drop-shaped paramedian osteoderm of *Prestosuchus chiniquensis* figured in Scheyer & Desojo (2011, pl.1, fig. 5).

Twelve osteoderms (MFSN 31568-69, 31582 and 46828-36) have a sub-quadrangular to pentagonal outline and a peculiar fingerprint-like sculpturing of the external surface (Figs. 5H–5L). These scale-like osteoderms are very thin (<1mm) and smaller than the paramedian osteoderms of MFSN 46485 (width ranges 10–12 mm and length 8–10 mm). Usually they are slightly arched. One further specimen (MFSN 46837) has the same sculpturing but a different L-shaped outline and is larger (23 mm × 13 mm). The internal surface is visible only in this latter specimen; it presents thin and spaced wrinkles that are unidirectionally oriented.

Usually the sub-quadrangular to pentagonal osteoderms (*e.g*., MFSN 46829, MFSN 31568, MFSN 46828, and MFSN 46833; Figs. 5H–5L) have a narrow unornamented band along one of the longer sides resembling the articular lamina of some archosaur osteoderms. The margin of one of the shorter sides perpendicular to that bearing the unornamented band is sometimes bent internally, whereas the other is flat (Figs. 5H–5I and 5K–5L); the bent side is often less ornamented than the rest of the osteoderm (Figs. 5H–5I and 5K) and may have been a zone of overlap with other osteoderms. If the side with the unornamented lamina is the anterior one, the complete osteoderms are usually wider than long, except MFSN 46833, which is as long as wide (Fig. 5L). The pentagonal outline of some specimens as MFSN 46828 and MFSN 46829 suggests that they interlocked with other similar osteoderms to form a compact structure (like, for example, the gular shield of phytosaurs; Long & Murry, 1995, fig. 46).

**PHYLOGENETIC ANALYSES**

**Figure 6:**
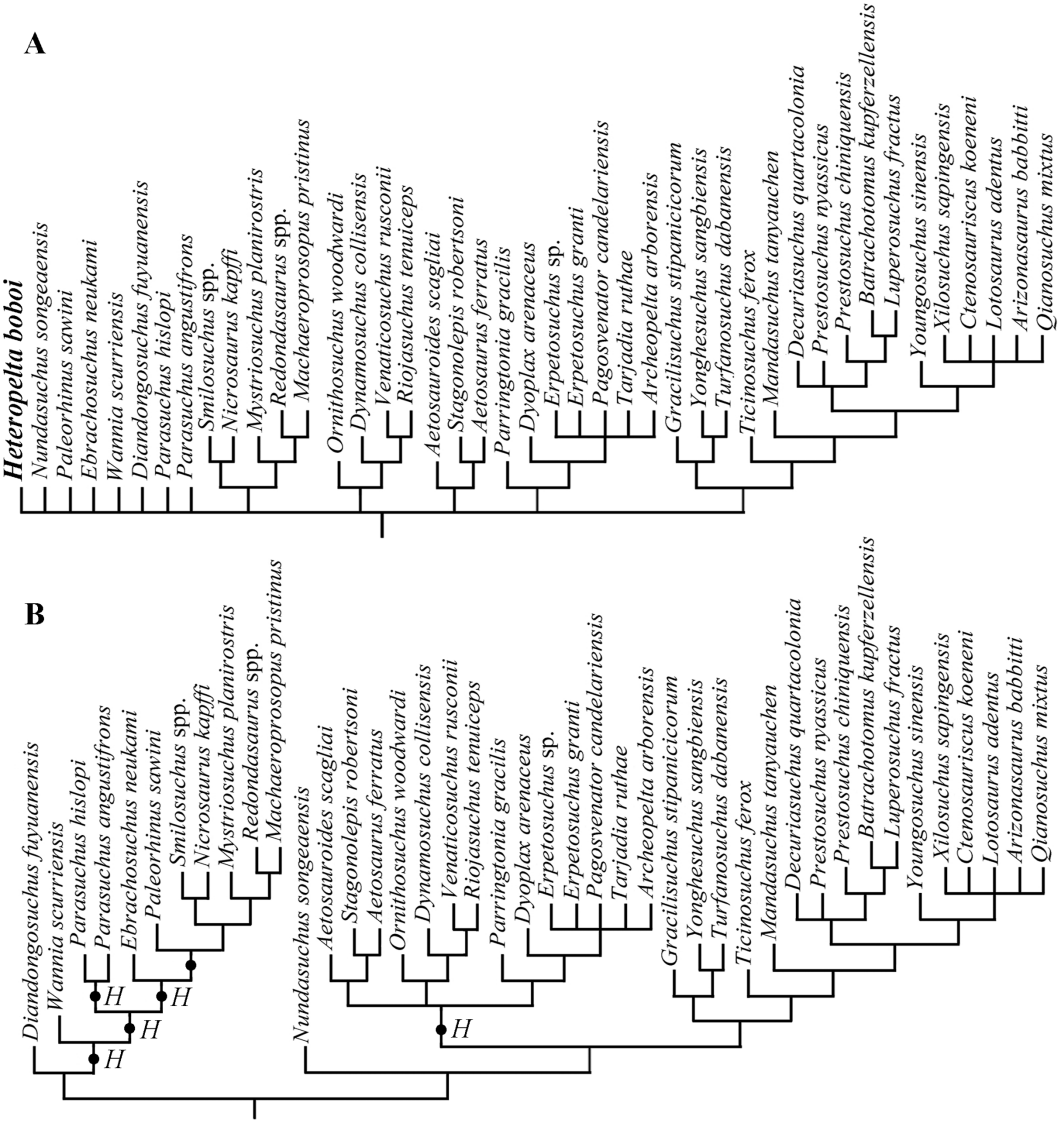
Phylogenetic position of *Heteropelta boboi* using the matrix by Ezcurra et al. (2020). (A) The pseudosuchian portion of the strict consensus tree of 3640 most parsimonious trees (tree length = 4999). (B) The alternative positions of *Heteropelta boboi* (indicated with *H*) in the most parsimonious trees.

Elaborating the matrix by Ezcurra et al. (2020), TNT found 3,640 most parsimonious trees (MPTs) each with a tree length of 4,999 steps, a consistency index (CI) of 0.2142 (CI excluding uninformative characters = 0.2125), and a retention index (RI) of 0.6503. *Heteropelta boboi* falls within the Pseudosuchia and its addition causes a loss of resolution within this clade in the strict consensus tree (Fig. 6A) relative to the strict consensus tree of Ezcurra et al. (2020, Extended Data fig. 4). Two main alternative positions are found for *H. boboi* by the analysis (Fig. 6B): (1) basal in the Phytosauria, as a basal member of the sister group of the basalmost phytosaur *Diandongosuchus fuyuanensis*, (2) basal in the Suchia as the sister taxon of a trichotomy of Aetosauria, Ornithosuchidae and Erpetosuchidae.

**Figure 7:**
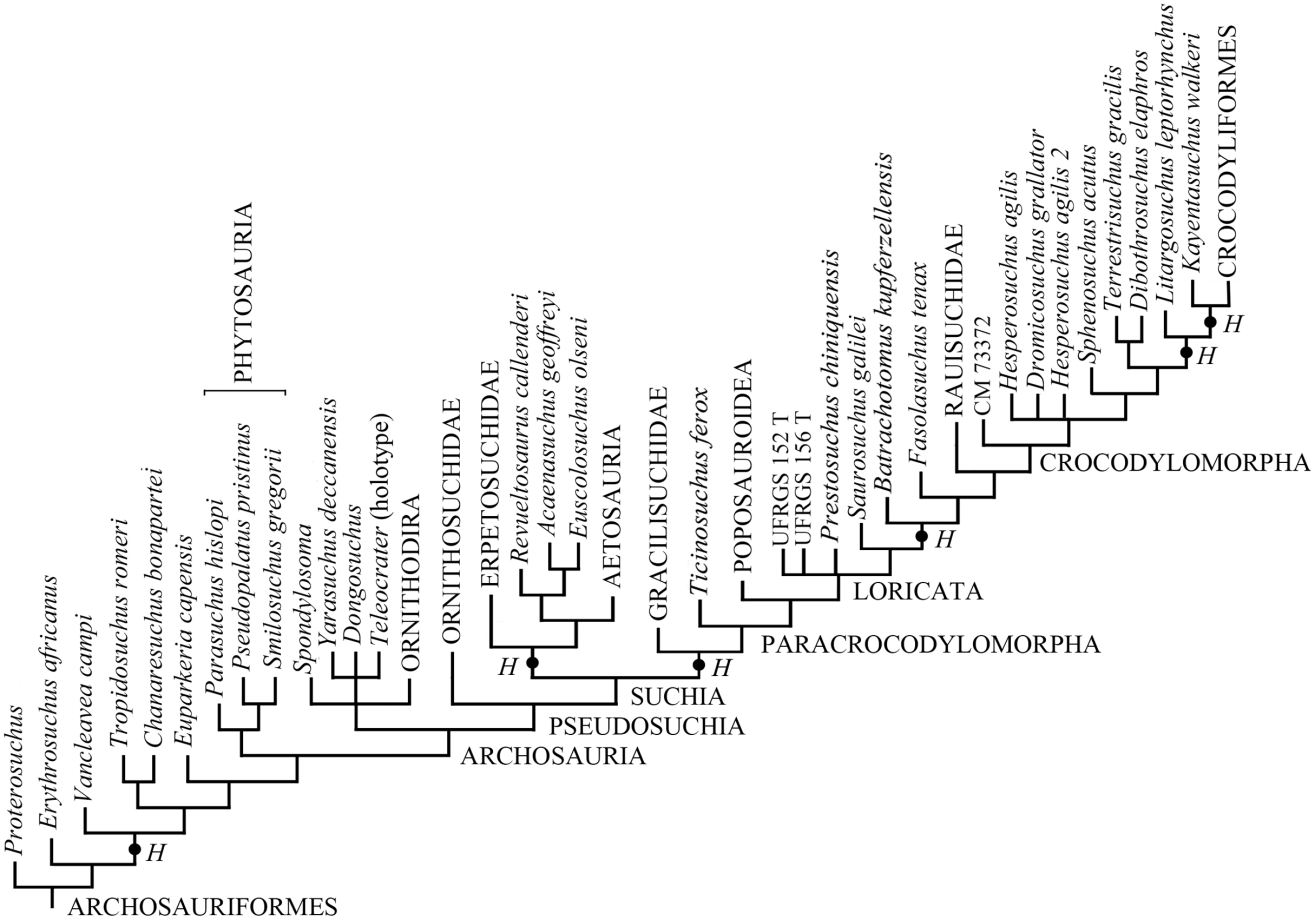
Phylogenetic position of *Heteropelta boboi* using the matrix by Marsh et al. (2020). The alternative positions of *Heteropelta boboi* (indicated with *H*) in the MPTs. Some terminal species-level taxa have been grouped into the corresponding supraspecific taxa for graphic reasons; *Mesosuchus browni*, *Prolacerta broomi*, and *Archosaurus russicus* (the sister-taxon of *Proterosuchus*), are not reported in the tree.

Elaborating the matrix by Marsh et al. (2020), TNT found 4,535 MPTs trees with a tree length of 1,407 steps, a CI of 0.3703 (CI excluding uninformative characters = 0.3649), and a RI of 0.7657. The addition of *H. boboi* causes a substantial loss of resolution within the Archosauriformes in the strict consensus tree (Fig. S1) with respect to the strict consensus tree obtained from the same matrix without the new taxon. Six alternative positions have been found for *H. boboi* by the analysis (Fig. 7): (1) basal in the Archosauriformes as the sister taxon of *Vancleavea campi* + (*Tropidosuchus romeri* + *Chanaresuchus bonapartei*) + *Euparkeria capensis* + Phytosauria + Archosauria; (2) basal in the Suchia as the sister taxon of Erpetosuchidae + (Aetosauria + (*Revueltosaurus callenderi* + (*Acaenasuchus geoffreyi* + *Euscolosaurus olseni*))); (3) basal in the Suchia as the sister taxon of Gracilisuchidae + Paracrocodylomorpha; (4) basal in the Loricata as the sister taxon of *Batrachotomus kupferzellensis* + *Fasolasuchus tenax* + Rauisuchidae + Crocodylomorpha; (5) within Crocodylomorpha as the sister taxon of *Litargosuchus leptorhynchus* + *Kayentasuchus walkeri* + Crocodyliformes; and (6) within Crocodylomorpha as the sister taxon of *Kayentasuchus walkeri* + Crocodyliformes.

## Discussion

Osteoderms occur in many tetrapods, including plagiosaurid, dissorophid and chronosuchian ‘amphibians’ (Witzmann & Soler-Gijón, 2010), synapsids (*e.g*., cingulate and mylodontid xenarthrans), parareptiles (Romer, 1956; Sues & Reisz, 2008), placodonts (Rieppel, 2000), *Eusaurosphargis* (Scheyer et al., 2017), saurosphargids (Li et al., 2011, 2014), and squamates (Romer, 1956; Kirby et al., 2020), but they are most common among archosauriform diapsids (Nesbitt, 2011; Ezcurra, 2016). Osteoderms occur dorsal to the neural spines of the presacral vertebrae in most archosauriforms (Nesbitt, 2011; Ezcurra, 2016). They are absent in poposauroid pseudosuchians (except the basal *Qianosuchus mixtus*) and the avian-line archosaurs plesiomorphically; they were recovered independently in some dinosaurs (*i.e.*, thyreophorans, some titanosaur sauropods and the theropod *Ceratosaurus*) (Nesbitt, 2011; Ezcurra, 2016). The morphology and arrangement of the osteoderms is often diagnostic at various taxonomic levels within archosauriforms (Nesbitt et al., 2009; Nesbitt, 2011; Desojo et al., 2013; Sues, Desojo & Ezcurra, 2013; Ezcurra, 2016).

Among the reptiles identified in the TAF, ichthyosaurs (McGowen & Motani, 2003), eusauropterygians (*sensu* Rieppel, 2000) and non-archosauriform archosauromorphs (Nesbitt, 2011; Ezcurra, 2016) lack osteoderms. Cyamodontoid placodonts have carapace-like armor made of coosified osteoderms (Rieppel, 2000). Skeletal elements belonging to the Anisian-Ladinian *Eusaurosphargis dalsassoi*, saurosphargids, and non-cyamodontoid placodonts have not been identified in the TAF yet. Furthermore, the relative size, shape and arrangement of the osteoderms in *Eusaurosphargis dalsassoi* (see Scheyer et al., 2017) and saurosphargids (Li et al., 2011, 2014) are unlike those of *H. boboi*. In particular, *Eusaurosphargis* and saurosphargids lack paramedian columns of imbricated osteoderms; saurosphargids have dorsal armor made mainly of elongated granular osteoderms. Non-cyamodontoid placodonts also lack paramedian columns of imbricated osteoderms; they have a single row of osteodems located on the apex of the neural spines of the vertebrae (Rieppel, 2000). *Pararcus diepenbroeki* possesses unfused osteoderms of different size and shape, but their arrangement is unknown (Klein & Scheyer, 2014).

The only other osteoderm-bearing tetrapods identified in the TAF are the archosauriforms, therefore *H. boboi* is plausibly an archosauriform reptile.

In addition to MFSN 46485 and the isolated osteoderms, the archosauriform remains include serrated teeth, cervical, dorsal and caudal vertebrae, a scapula, an ischium, and two calcanei (Dalla Vecchia, 2006, 2008; Dalla Vecchia & Simonetto, 2018). Because these elements do not have any shared morphology with the holotype of *H. boboi*, are isolated and scattered, do not come from the same bed as the holotype, and most of them are not even from the same outcrop, they cannot be unambiguously referred to this taxon, although at least some of them may belong to it. They will be described elsewhere.

The knowledge on the actual morphology and extent of the dermal armor of extinct archosauriforms is hampered by the typical disarticulation of their skeletons, including the osteoderms. MFSN 46485 is exceptional in preserving several columns of osteoderms still in their anatomical arrangement.

### The armor fragment: anatomical position and orientation

The larger osteoderms of *Heteropelta boboi* that are arranged on two paramedian columns resemble those of the dorsal paramedian osteoderms of many pseudosuchian archosaurs like ornithosuchids (Walker, 1964, fig. 14), aetosaurs (Long & Murry, 1995), erpetosuchids (Benton & Walker, 2002, figs. 3–4 and 7–8), gracilisuchids (Lecuona, 2013, figs. 3.51–55), *Ticinosuchus ferox* (see Krebs, 1965, fig. 61), ‘rauisuchians’ (*e.g*., *Batrachotomus kupferzellensis*, Gower & Schoch, 2009, fig. 1M; *Saurosuchus galilei*, Trotteyn, Desojo & Alcober, 2011, fig. 10; *Prestosuchus ciniquensis*, Roberto-Da-Silva et al., 2020, fig. 6A), and basal crocodylomorphs (Brown, 1933, fig. 1; Colbert & Mook, 1951, fig. 9b; Nash, 1975, fig. 18; Clark, Sues & Berman, 2000, figs. 1 and 3B; Sues et al., 2003, figs. 2 and 4).

Armor made of osteoderms arranged in multiple columns may be present both dorsally and ventrally in armored archosauriforms. Abdominal armor occurs in *Vancleavea campi* and *Litorosuchus somnii*, *Doswellia kaltenbachi*, some aetosaurs, *Revueltosaurus callenderi*, *Protosuchus richardsoni* and some other crocodyliforms (Nesbitt, 2011; Ezcurra, 2016; Parker et al., 2021). However, the ventral armor is usually made of osteoderms with similar size and often fitting in a mosaic-like pattern, whereas paramedian columns of larger osteoderms are absent (Colbert & Mook, 1951; Nesbitt et al., 2009; Ezcurra, 2016; Parker et al., 2021). Phytosaurs have a unique ‘gular shield’ composed of multiple osteoderms overlapping and interlocking under the throat which lacks paramedian columns of larger osteoderms (Stocker & Butler, 2013).

The dorsal position of the osteoderm columns with respect to the neural arches and the presence of paramedian columns of larger osteoderms, suggests that MFSN 46485 is a dorsolateral fragment of armor. The position of this fragment in the body of *H. boboi* could be established only by observing the features of the neural arches. The presence of transverse processes projecting laterally from the arches and the relative gracility and curvature of the purported rib remains suggest that the fragment is from the dorsal region of the vertebral column. As indicated above, it is from the right side of the body based on the zygapophyseal articulation and inferred anteroposterior polarity.

### Comparison of MFSN 46485 with the isolated osteoderms

The isolated square osteoderms are similar to the paramedian osteoderms of MFSN 46485 in their overall shape and size, coarse and irregular ornamentation, and probably in the kind of articulation, but differ in details. They do not show the asymmetry between the medial and lateral part of the paramedians of MFSN 46485. The keel appears to be blunter and the ornamentation of the external surface is slightly different: there are no branching ridges perpendicular to the keel, no smooth areas, and no asymmetry of the ornamentation in the two halves. Furthermore, there is never a notch in correspondence of the end of the keel on the side opposite to that bearing the median process.

MFSN 46618 resembles in shape and size the most lateral osteoderms (lateral column 6) of MFSN 46485. Unfortunately, the poor conditions of the latter do not allow for further comparison.

In some archosauriforms (*e.g., Vancleavea campi*, Nesbitt et al., 2009; *Litorosuchus somnii*, Li et al., 2016; *Doswellia kaltenbachi*, Dilkes & Sues, 2009; aetosaurs, Desojo et al., 2013; *Ticinosuchus ferox*, Krebs, 1965, fig. 61; *Prestosuchus ciniquensis*, Roberto-Da-Silva et al., 2020, p. 12; *Postosuchus alisonae*, Peyer et al., 2008, fig. 6) the shape of the osteoderms changes based on the region of the body that they cover and even within each region. This means that the isolated osteoderms from the TAF may belong to *Heteropelta boboi* but are different from those of MFSN 46485 because they are from different regions of the body. Furthermore, intraspecific variation in osteoderm shape and distribution may be possible (English, 2018). However, two facts suggest some caution in referring the isolated osteoderms to *H. boboi*. The first is that no small isolated lateral osteoderm has ever been found. This could be due to taphonomic factors and be a consequence of selective transport, but it may also mean that *H. boboi* was rare in the TAF. The second fact is that several archosauriform vertebrae clearly different from those of MFSN 46485 occur in the Aupa sample (Dalla Vecchia, 2008, figs. 72B and F), including cervicals, dorsals and caudals, whereas vertebrae with neural arches like those of MFSN 46485 have never been found. This means that different archosauriforms are present in the sample and support the hypotheses that *H. boboi* may be rare in the formation and that many of the isolated osteoderms may not pertain to *H. boboi*. This aspect can be better understood only by preparation and study of the other archosauriform material from the TAF and discovery of additional material.

The smaller and thinner osteoderms with fingerprint-like ornamentation are very different from both the osteoderms of MFSN 46485 and from the larger isolated osteoderms. However, it cannot be excluded that they formed the ventral armor of the same animal, a gular shield like that of the phytosaurs, or rows along the limbs. Appendicular osteoderms are present in *Vancleavea campi* and *Litorosuchus somnii*, doswelliids, phytosaurs, aetosaurs, erpetosuchids (*Tarjadia ruthae*), and some crocodylomorphs (Nesbitt, 2011; Stocker & Butler, 2013; Ezcurra, 2016). Alternatively, they might be fish scales. However, they do not show the growth lines that are typical of fish scales. Furthermore, the only fish remains found associated with the reptile bones from the TAF are two skull bones of a large coelacanthiform and the osteoderms with fingerprint-like ornamentation do not resemble the scales of the coelacanthiforms (Martín-Abad et al., 2017).

### Systematic comparison

The only Triassic archosauriforms with more than two columns of dorsolateral osteoderms per side in the trunk are the early Norian *Vancleavea campi* and its Ladinian sister taxon *Litorosuchus somnii* and the late Ladinian-Norian Doswelliidae (Sues et al., 2003; Nesbitt et al., 2009; Nesbitt, 2011; Ezcurra, 2016; Li et al., 2016; Ezcurra et al., 2020).

*Euparkeria capensis* has two columns of paramedian osteoderms plus an outer row of much smaller osteoderms which are not in contact with each other (Ewer, 1965, p. 415). In the Proterochampsidae, osteoderms occur dorsal to the neural spines of the postaxial vertebrae arranged in a single median column (Nesbitt, 2011; Ezcurra, 2016). Within crocodile-line archosaurs, Ornithosuchidae, Gracilisuchidae, *Ticinosuchus ferox* and Loricata (’rauisuchians’ and basal crocodylomorphs) are reported to have only the two columns of paramedian osteoderms, whereas some phytosaurs, the aetosaurs, *Revueltosaurus callenderi*, and the erpetosuchids have an additional column of lateral osteoderms one each side, and these are usually smaller (not as wide as) than the paramedians (Nesbitt, 2011; Bader & Stocker, 2014; Ezcurra, 2016; Ezcurra et al., 2017; Parker et al., 2021).

In *Vancleavea campi*, the entire body is covered by osteoderms. *Vancleavea* lacks columns of distinct paramedian osteoderms that are much larger than the other osteoderms; furthermore, its osteoderms are externally smooth. Dorsal and lateral osteoderms of the trunk are poorly exposed; the other osteoderms have rather different shapes with respect to those of *H. boboi* (see Nesbitt et al., 2009, figs. 18-19). The trunk of the closely related *Litorosuchus somnii* presents many large and rounded osteoderms, but it is unclear whether it is entirely covered by them or not and whether all dorsolateral osteoderms are arranged in columns or not (Li et al., 2016, fig. 1a-b, d). The dorsolateral osteoderms of *Litorosuchus somnii* are unlike those of *H. boboi*. Those along the dorsal midline are apparently arranged in paramedian columns, but are roundish, with convex anterior margin, concave posterior edge, a symmetrical development with respect to the longitudinal median ridge, faint ornamentation of very thin radial ridges that become broader close to the margin of the osteoderm, which is irregular and nearly serrated, and are not imbricated (Li et al., 2016, fig. 6e). The purported lateral osteoderms are oval or nearly round in outline, with a small projection on the anterior margin, a midline ridge but no other ornamentation, and are as large as the dorsal osteoderms (Li et al., 2016, fig. 1d). Furthermore, *Litorosuchus* has comparatively broader neural spines and shorter postzygapophyses in the dorsal vertebrae than *H. boboi*.

Doswelliids preserve multiple columns and rows of osteoderms. *Doswellia kaltenbachi* has at least 10 columns of osteoderms (five per side) in the posterior dorsal region (Sues, Desojo & Ezcurra, 2013). According to Ezcurra (2016, p. 296), “more than two rows [= columns] of dorsal osteoderms” is a diagnostic feature of the Doswelliidae. Unlike *Heteropelta boboi*, the osteoderms of the doswelliids posterior to the “nuchal collar” (Weems, 1980, fig. 23) are all square or rectangular in shape and of similar size and possess a characteristic ornamentation made of deep subcircular pits with subequal size (Dilkes & Sues, 2009, figs. 9–11; Schoch & Sues, 2014, figs. 1 and 5). The peripheral pits of the osteoderms are groove-like and externally open. The external side of the osteoderm has a median blunt keel in *Doswellia kaltenbachi* and *Jaxtasuchus salomoni*, whereas it bears a spike-like dorsal eminence projecting outwardly from the midpoint in *Rugarhynchus sixmilensis* (see Wynd et al., 2020). Shape and ornamentation of the paramedian osteoderms of the erpetosuchids *Tarjadia ruthae* (Ladinian-Carnian boundary), *Archeopelta arborensis* (Ladinian-Carnian) and *Parringtonia gracilis* (Anisian) are similar to those of doswelliids, but the osteoderms are thicker in the erpetosuchids (Ezcurra et al., 2017).

Doswelliids and erpetosuchids (Ezcurra et al., 2017) do not have a point-and-notch articulation of the osteoderms, but an overlap along an anterior lamina, a structure devoid of ornamentations, that is thinner than the rest of the dermal plate (this structure occurs in the Aupa valley osteoderms with fingerprint-like ornamentation, Figs. 5H–5L). In *Doswellia kaltenbachi*, this anterior smooth bar is separated from the pitted portion of the osteoderm by a groove. The osteoderms of the Norian *Revueltosaurus callenderi* (see Parker et al., 2021), the Carnian *Euscolosuchus olseni* (see Sues, 1992), and many aetosaurs also have this kind of articulation but the unornamented articular portion is typically formed by a raised bar (Desojo et al., 2013).

The dorsal armor of the phytosaurs is poorly known and its morphological variation has been largely overlooked (Bader & Stocker, 2014). According to Stocker & Butler (2013, p. 91), the dorsal armor of phytosaurs consists only of two paramedian columns of “diamond-shaped or roughly triangular osteoderms” that often “have dorsal surfaces covered with patterns of ridges and grooves surrounding a central keel”. Osteoderms that are rounder than the paramedians loosely cover the appendicular skeleton (Stocker & Butler, 2013). However, Bader & Stocker (2014) have later reported the presence of a column of paramedians and a column of lateral osteoderms per side in the trunk of *Angistorhinus alticephalus*. Associated paramedian and lateral osteoderms have been described in phytosaurs also by Chatterjee (1978) and Long & Murry (1995). According to Gozzi & Renesto (2003), *Mystriosuchus planirostris* has four columns of osteoderms in the trunk, a paramedian and a lateral column per side. Shape and sculpture of the osteoderms are variable within the phytosaur species. Long & Murry (1995, p. 34) described the paramedian osteoderms as “tongue-shaped”, while those of *Redondasaurus* are “polygonal” (Spielmann & Lucas, 2012, p. 63). The keel is actually a posterior bulge in the trunk paramedians of *Parasuchus hislopi* figured by Chatterjee (1978, fig. 9b–i). The paramedians of *Mystriosuchus planirostris* are semicircular in dorsal view, while the lateral osteoderms are elliptical with a longitudinal median keel (Gozzi & Renesto, 2003, fig. 15); they have similar sizes. The paramedians of some phytosaurs articulate with the preceding osteoderms along the apparently smooth anterior edge (Long & Murry, 1995, fig. 44; Spielmann & Lucas, 2012, p. 63) or are imbricated with the anterior osteoderm overlapping the posterior (Chatterjee, 1978, fig. 9b–c). The trunk paramedians of *Angistorhinus alticephalus* overlap the anterior edges of the succeeding osteoderms, but there are no articular facets (Bader & Stocker, 2014); the lateral osteoderms are not much smaller than the paramedians, are variable in shape, and “have anteroposteriorly-directed, elongate dorsal keels that are undercut medially by deep fossae” (Bader & Stocker, 2014, p. 83). The paramedians of *Mystriosuchus planirostris* articulated medially along the straight margin, which is bordered laterally by a longitudinal “carina” (Gozzi & Renesto, 2003, fig. 15). The trunk osteoderms of the phytosaurs do not share any diagnostic feature with those of *H. boboi*. Furthermore, phytosaurs have comparatively broader neural spines and shorter postzygapophyses in the dorsal vertebrae than *H. boboi* (Chatterjee, 1978; Long & Murry, 1995; Gozzi & Renesto, 2003; Spielmann & Lucas, 2012).

Trunk paramedians of the aetosaurs are characteristically quadrangular, much wider than long (width:length ratios of approximately 2.5–4:1) and usually have a dorsal eminence and radial ornamentation of pits, ridges and grooves; lateral osteoderms are smaller and narrower than the paramedians and bear eminences that can enlarge into spines or horns (Desojo et al., 2013). The trunk paramedians of *Revueltosaurus callenderi* are similar to those of the aetosaurs (Parker et al., 2021). The trunk osteoderms of *Acaenasuchus geoffreyi* are also similar to those of the aetosaurs (Marsh et al., 2020). The paramedians of *Euscolosuchus olseni* are sharply angulated and bear greatly enlarged lateral spines (Sues, 1992, figs. 1 and 3).

The paramedians of the ornithosuchids have simple ornamentation and bear a dorsolateral prominence on their external surfaces (von Baczko & Ezcurra, 2013). The external surface of the anterior part of the trunk paramedians of *Ornithosuchus longidens* presents marked radial ridges where the preceding osteoderm overlaps it (Walker, 1964, fig. 14i).

Trunk paramedians of the Gracilisuchidae are imbricated, have an approximately square outline and an anterior process, are slightly asymmetrical with a longitudinal ridge that is offset medially from the midline, and have a distinct, longitudinal bend near the lateral edge (Lecuona, 2013; Butler et al., 2014). There are two osteoderms per vertebra. The dorsal vertebrae of the gracilisuchids have broad and fan-shaped neural spine in lateral view (Ezcurra, 2016).

The same kind of imbrication of *H. boboi* paramedian and lateral osteoderms occurs in the paramedians of the euparkeriid *Halazhaisuchus qiaoensis* (see Wu, 1982, fig. 1), the basal pseudosuchian *Nundasuchus songeaensis* (see Nesbitt et al., 2014, fig. 6A–B), the gracilisuchid *Gracilisuchus stipanicicorum* (see Lecuona, 2013, figs. 3.51–52, 54–55), and the paracrocodylomorphs *Ticinosuchus ferox* (see Krebs, 1965, figs. 8 and 61), *Mandasuchus tanyauchen* (see Butler et al., 2018), *Saurosuchus galilei* (see Sill, 1974, fig. 10), *Prestosuchus ciniquensis* (see Desojo, von Baczko & Rauhut, 2020), *Decuriasuchus quartacolonia* (França, Ferigolo & Langer, 2011, fig. 3B), and *Batrachotomus kupferzellensis* (see Gower & Schoch, 2009, fig. 1). However, the polarity of imbrication in all of these taxa is reversed with respect to the Friulian taxon: the anterior osteoderm overlaps the posterior one and the median pointed process is anterior.

The paramedian osteoderms of the coeval (Anisian/Ladinian) *Ticinosuchus ferox* are characteristically arrowhead-shaped and symmetrical (Krebs, 1965; Lautenschlager & Desojo, 2011), rather unlike those of *H. boboi*.

An asymmetrical development of the medial and lateral part of the paramedians similar to that of MFSN 46485 is observed in the paramedians of the doswelliid *Jaxtasuchus salomoni* (see Wynd et al., 2020, fig. 2B–C), in the cervical osteoderms of the erpetosuchid *Erpetosuchus granti* (see Benton & Walker, 2002, fig. 8), and in the trunk paramedians of the loricatan *Batrachotomus kupferzellensis* (see Gower & Schoch, 2009, p. 117) and *Postosuchus alisonae* (see Peyer et al., 2008, fig. 6B–D).

In *Rauisuchus tiradentes* (see Lautenschlager & Rauhut, 2015, fig. 16A–C) and *Postosuchus* spp. (Peyer et al., 2008; Weinbaum, 2013), the articular pointed process is displaced medially, whereas it is displaced laterally in some basal crocodylomorphs (*e.g*., *Dromicosuchus grallator*, Sues et al., 2003 and those with the anterior smooth bar mentioned below).

At least some basal crocodylomorphs have both the point-and-notch articulation and the smooth anterior lamina in the paramedians (*e.g., Hesperosuchus agilis*, Colbert, 1952, fig. 33; *Orthosuchus stormbergi*, Nash, 1975, fig. 18), but the pointed anterior process, which is the continuation of the longitudinal keel, is asymmetrically placed closer to the lateral margin of the osteoderm, because the portion lateral to the keel is much narrower than the medial one, unlike *H. boboi*; furthermore the lateral portion of the osteoderm is angled with respect to the medial one, unlike the paramedians of *H. boboi*.

### Phylogenetic analysis

The analysis carried out using the matrix of Ezcurra et al. (2020) restricts the affinity of *H. boboi* within the pseudosuchians as either a basal phytosaur or a basal suchian.

*H. boboi* shares with the sister group of the basalmost phytosaur *Diandongosuchus fuyuanensis* the following non-ambiguous synapomorphies: “dorsal osteoderms present, with more than two rows” (587.3, ordered) and “sculpture on the external surface of the osteoderms present” (588.1). The other positions within the Phytosauria found by the analysis are not supported by non-ambiguous synapomorphies. No osteoderm character is scored for *Wannia scurrensis*, *Parasuchus angustifrons*, *Ebrachosuchus neukami*, *Paleorhinus sawini*, *Nicrosaurus kapffi*, and *Redondasaurus* spp. Note that the doswelliids are also scored 3 and 1 for characters 587 and 588, respectively.

Character states 587.3 and 588.1 are non-ambiguous synapomorphies shared by *H. boboi* + (Aetosauria + Erpetosuchidae) and *H. boboi* + (Aetosauria + (Ornithosuchidae + Erpetosuchidae)) too. Aetosauria + (Ornithosuchidae + Erpetosuchidae) share with *H. boboi* the following further non-ambiguous synapomorphies: “relation between paramedian dorsal osteoderms and presacral vertebrae one to one” (592.0), “dorsal osteoderm alignment dorsal to the dorsal vertebrae one to one” (593.1), and “presacral paramedian osteoderms without a distinct longitudinal bend near the lateral edge” (597.0). Thus, relationships of *H. boboi* with basal suchians are more supported than with phytosaurs.

The analysis based on the matrix by Marsh et al. (2020) does not find support for a close relationship of *H. boboi* with phytosaurs. Instead, it suggests possible relationships with nearly all other armored archosauriforms included in the analysis: basal archosauriforms, basal suchians, basal loricatans and crocodylomorphs.

This uncertainty in the phylogenetic relationships of *H. boboi* can be plausibly explained with its peculiarity, the presence of features shared with different clades of armored archosauriforms, and the incompleteness of the only known specimen. These alternative relationships of the Italian taxon will likely be resolved only by finding cranial and postcranial elements associated with armor remains, as happened to *Acaenasuchus geoffreyi* (see Marsh et al., 2020).

## Conclusions

MFSN 46485 is comprised of a dorso-lateral fragment of armor with a row of neural arches of the dorsal vertebrae. The armor is composed of two columns of paramedian osteoderms and six columns of lateral osteoderms from the right side of the body. It is an uncommon case of articulated dorsal armor comprised of non-coosified osteoderms. Unlike other armored archosaurs, the osteoderms are imbricated with the posterior osteoderm overlapping the anterior one, as indicated by the orientation of the zygapophyseal articulation of the neural arches. The neural arches are low, with small neural spines and long postzygapophyses. The osteoderms of the lateral columns increase in size and change in shape from the most medial to the most lateral columns. The specimen is the holotype of the new taxon *Heteropelta boboi*. Among the reptile taxa represented by remains found in the same locality and horizon, the new taxon can be referred only to the Archosauriformes. Among the latter, only the non-archosaur proterochampsians *Vancleavea campi* and *Litorosuchus somnii* and the doswelliids have a dorsal armor comprised of more than two columns of osteoderms per side, but the morphology and arrangement of their osteoderms is unlike those of *H. boboi*. When added to the matrix of Ezcurra et al. (2020), the new taxon falls within the Pseudosuchia, alternatively as a phytosaur or basal in the Suchia as the sister taxon of a trichotomy of Aetosauria, Ornithosuchidae and Erpetosuchidae; when added to the matrix of Marsh et al. (2020), it results alternatively as a basal archosauriform, basal suchian, basal loricatan or crocodylomorph. Therefore, its affinity is uncertain. However, the armor of *H. boboi* is unique within the Archosauriformes and the phylogenetic relationships of the species will be resolved only with the discovery of cranial and postcranial remains associated with its diagnostic armor elements.

The life environment of *H. boboi* may have been the close emergent land of the Anisian Paleocarnic Ridge or the sea bordering it (as suggested by the associated coastal reptiles). Thus, the new armored reptile may have been a semi-aquatic archosauriform like *Vancleavea campi* and related taxa (Li et al., 2016), the doswelliids (Sues, Desojo & Ezcurra, 2013) and phytosaurs (Stocker & Butler, 2013), but fully terrestrial habits cannot be excluded.

## Supplemental Information

10.7717/peerj.12468/supp-1 Supplemental Information 1 The nexus matrix by Ezcurra et al. (2020) with the addition of *Heteropelta boboi*.

10.7717/peerj.12468/supp-2 Supplemental Information 2 Data matrix by Marsh et al. (2020). The scores of *Heteropelta boboi* that have been added to the matrix by Marsh et al. (2020).

10.7717/peerj.12468/supp-3 Supplemental Information 3 Phylogenetic position of *Heteropelta boboi* using the matrix by Marsh et al. (2020). The strict consensus tree of 4,536 MPTs.

## Institutional abbreviations

MFSN: Museo Friulano di Storia Naturale, Udine, Italy.

## Additional Information and Declarations

### Data Availability

The following information was supplied regarding data availability: This is a paleontological paper without raw data or code. The specimen is stored at Museo Friulano di Storia Naturale (Friulian Museum of Natural History): MFSN 46485.

### New Species Registration

The following information was supplied regarding the registration of a newly described species: Publication LSID: urn:lsid:zoobank.org:pub:B91F17BF-7B38-4034-B761-170916EBA16E *Heteropelta*: urn:lsid:zoobank.org:act:8F8B8564-B2E6-4B7D-B2E1-14BDFAB36900 *Heteropelta boboi*: urn:lsid:zoobank.org:act:BCADA4F4-47FA-448B-B71D-6D733AD3AACE.
