# Supplementary material for: Heteropelta boboi n. gen., n. sp. an armored archosauriform (Reptilia: Archosauromorpha) from the Middle Triassic of Italy
Source: PeerJ. 2021 Nov 15;9:e12468. doi: 10.7717/peerj.12468 (PMC8601055; doi:10.7717/peerj.12468)
Supplement: Supplemental Information 2 — The scores of Heteropelta boboi that have been added to the matrix by Marsh et al. (2020). [file peerj-09-12468-s002.docx]

The scores of *Heteropelta boboi* that have been added to the matrix by Marsh et al. (2020)*.*

?????????? ?????????? ?????????? ?????????? ?????????? ?????????? ?????????? ?????????? ?????????? ?????????? ?????????? ?????????? ?????????? ?????????? ?????????? ?????????? ?????????? ?????????? ?????????? ?????????? ?????????? ?????????? ?????????? ?????????? ?????????? ?????????? ?????????? ?????????? ?????????? ?????????? ?????????? ?????????? ?????????? ?????????? ?????????? ?????????? ?????????? ?????????? ?????????? ?????????? 1110??00?0 1????????- -------1?? ----?-???? -???-
