# Supplementary figures and images for: Heteropelta boboi n. gen., n. sp. an armored archosauriform (Reptilia: Archosauromorpha) from the Middle Triassic of Italy
Source: PeerJ. 2021 Nov 15;9:e12468. doi: 10.7717/peerj.12468 (PMC8601055; doi:10.7717/peerj.12468)

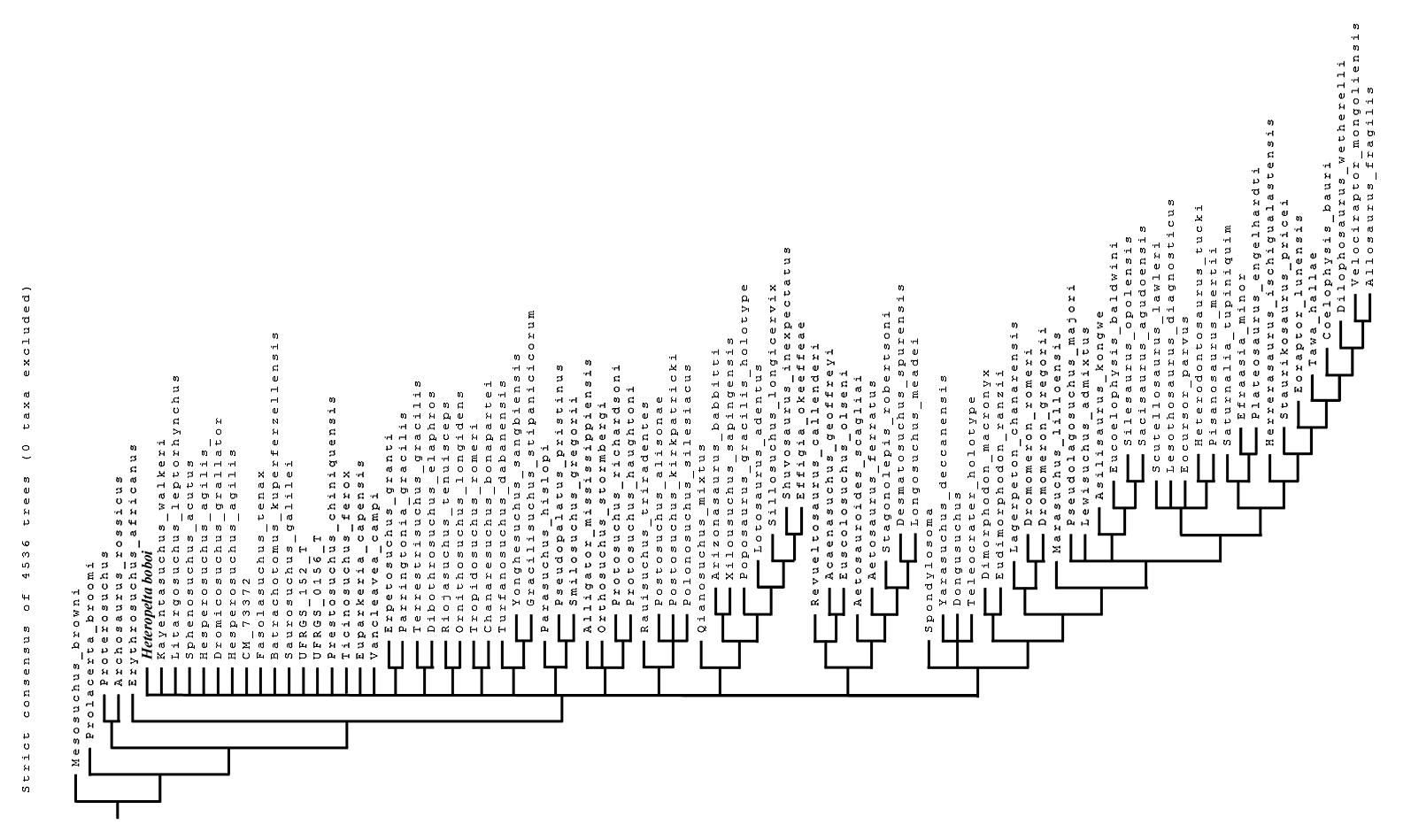

Supplement: Supplemental Information 3 — The strict consensus tree of 4,536 MPTs. [file peerj-09-12468-s003.png]
